# Taxonomic Implications of Leaf Micromorphology Using Microscopic Analysis: A Tool for Identification and Authentication of Korean Piperales

**DOI:** 10.3390/plants9050566

**Published:** 2020-04-29

**Authors:** Jun-Ho Song, Sungyu Yang, Goya Choi

**Affiliations:** Herbal Medicine Resources Research Center, Korea Institute of Oriental Medicine, Naju 58245, Korea; songjh@kiom.re.kr (J.-H.S.); sgyang81@kiom.re.kr (S.Y.)

**Keywords:** *Asarum*, herbal medicine, leaf epidermis, *Piper*, scanning electron microscopy, stomatal index, trichomes

## Abstract

A comparative study of the leaf micromorphology of Korean Piperales, including medicinal materials, was performed through light microscopy and scanning electron microscopy, to evaluate their taxonomic significance. Piperales possessed both amphistomatic and hypostomatic leaves. The epidermal area ranged from 38 to 5077 μm^2^, and the stomatal area ranged from 201 to 2129 μm^2^. The stomatal index on the abaxial surface was higher than that on the abaxial surface. Anomocytic stomata occurred most commonly, but actinocytic, anisocytic, tetracytic, and staurocytic stomata were also found in certain taxa. Secretory idioblasts were found on all taxa studied except *Aristolochia*. Three main types of trichomes were defined—(1) glandular trichome; (2) simple multicellular trichome; and (3) two-armed multicellular Y-shaped trichome. Although the quantitative data on its own had somewhat limited taxonomic value, the various qualitative characteristics (e.g., epidermal surfaces, stomata types and positions, trichome types and density, and secretory idioblast types) had great taxonomic value. These characteristics might be taxonomically relevant and useful for developing an identification key. Additionally, we evaluated and supported the previous taxonomic system of Korean *Asarum*, using leaf micromorphological characteristics. Finally, through the application for authentication of herbal medicine, we revealed that leaf micromorphological characteristics can be used for accurate authentication.

## 1. Introduction

Various leaf characteristics have been widely used as diagnostic characteristics [1]. Especially, previous works have shown that leaf micromorphological (cuticular, epidermal) characteristics are more taxonomically useful than gross or external morphology [1,2]. Micromorphological characteristics of leaves have been applied in systematic studies for different taxonomic groups such as bryophytes [3,4], ferns [5,6,7], gymnosperms [8,9,10,11], and angiosperms [12,13,14,15]. Recently, leaf micromorphology using microscopic analysis has also been used to facilitate accurate authentication and quality control of medicinal plants [16,17,18,19,20,21]. Although leaf micromorphological characteristics are commonly used in the identification and authentication of plants, no comprehensive studies have investigated the leaf micromorphology of the order Piperales, which contains many important medicinal herbs.

The order Piperales Bercht. and J. Presl is placed in the clade magnoliids and consists of three families—Aristolochiaceae Juss., Piperaceae Giseke, and Saururaceae Martynov. [22]. This order comprises more than 4300 species and is the most diverse within the magnoliids, from a morphological point of view (including growth forms) [23]. Moreover, some members of the Piperales are economically important (including medicinal plants), e.g., *Aristolochia* L., *Asarum* L., *Piper* L., *Saururus* L., and *Thottea* Rottb. [24,25,26]. Additionally, secondary metabolites, like aristolochic acids and their derivatives, are notable owing to concerns about their toxicity and safety [27,28].

In Korea, Piperales includes 9–13 species representing five genera (*Aristolochia*, *Asarum*, *Houttuynia* Thunb., *Piper*, and *Saururus*), and all three families [29]. The Korean Herbal Pharmacopoeia have designated the roots and rhizomes of *Asarum* (medicinal name—‘Asiasari Radix et Rhizoma’), the vines of *Piper kadsura* (Choisy) Ohwi (medicinal name—‘Piperis Kadsurae Caulis’), the herbs of *Houttuynia cordata* Thunb. (medicinal name: ‘Houttuyniae Herba’), and *Saururus chinensis* (Lour.) Baill. (medicinal name: ‘Saururi Herba’), as herbal medicines [30,31].

Among the five Korean genera, there is some debate about the delimitation and identification of species within the genus *Asarum*, which is the largest genus in this group. Different species delimitations have been argued by different authors. According to Lee, T.B. [32,33] and Lee, W.T. [34], two *Asarum* species occur in Korea. However, So and Kim [35] recognized three species and seven forms from Korea. In a recent treatment in the ‘Flora of Korea’ [36], the genus *Asarum* consisted of four species. In a comprehensive treatment by Oh [37], seven species were recognized to be present in Korea. Various studies have investigated different aspects of *Asarum*, such as cytology [38], phylogeny [39], population genetics [40], and floral micromorphology [41]. However, to date there has been no study of the leaf micromorphology and their taxonomic implications, in a broad sampling of *Asarum*.

Although leaf micromorphology has received less attention than other reproductive characteristics (e.g., floral, fruits, and seeds) as a taxonomically informative characteristic, it has been demonstrated that epidermal cells, stomatal complex [13,42,43,44,45], trichomes [46,47,48,49,50], and epicuticular waxes [51,52,53,54] in the epidermal surface of the leaves are useful diagnostic and taxonomic characteristics.

The objectives of this study were to—(1) document and illustrate a detailed description of leaf micromorphology in Korean Piperales using light microscopy (LM) and field emission scanning electron microscopy (FE-SEM); (2) evaluate the taxonomic or diagnostic importance of leaf micromorphological features; and (3) apply the authentication by comparing the collective materials in natural populations and commercially distributed medicinal materials in herbal markets, in order to ensure quality controls.

## 2. Results

### 2.1. Epidermal Cells

**Figure 1:**
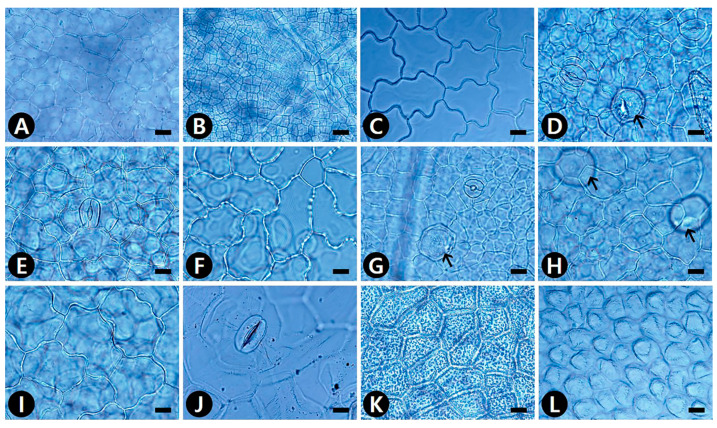
Light microscopy (LM) micrographs of adaxial (AD) leaf surfaces in Korean Piperales. (**A**) *Aristolochia contorta*, straight to curved anticlinal walls (ACW). (**B**) *A. manshuriensis*, straight ACW. (**C**) *Asarum heterotropoides* var. *mandshuricum*, undulous ACW. (**D**) *A. heterotropoides* var. *seoulense*, ano with idioblast (black arrow). (**E**) *A. koreanum*, sta. (**F**) *A. maculatum*, undulous ACW, striate, and wrinkled fine relief (FR). (**G**,**H**) *A. misandrum*, ano with idioblast (black arrow). (**I**) *A. versicolor*, straight to curved ACW. (**J**) *Houttuynia cordata*, sta. (**K**) *Piper kadsura*, straight ACW, tuberculate FR, and thick cell walls. (**L**) *Saururus chinensis*, straight ACW, conical periclinal cell wall (PCW). ano, anomocytic stomata; sta, staurocytic stomata. Scale bars = 20 μm.

**Figure 2:**
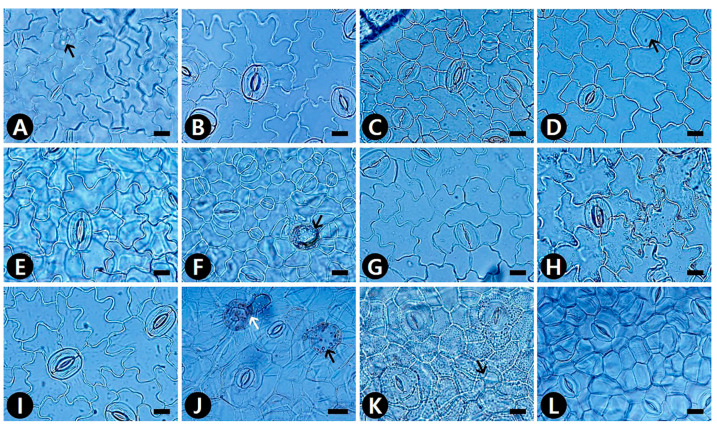
LM micrographs of abaxial (AB) leaf surfaces in Korean Piperales. (**A**) *Aristolochia contorta*, ano with idioblast (black arrow), lip-shaped, and thick and wide outer stomatal ledge aperture. (**B**) *Asarum heterotropoides* var. *mandshuricum*. (**C**) *A. heterotropoides* var. *seoulense*. (B–C) ano with sinuous ACW, double semicircle or lip-shaped and narrowly elliptical ledge aperture. (**D**) *A. koreanum*, sta with idioblast (black arrow). (**E**) *A. maculatum*, ano with sinuous ACW. (**F**) *A. misandrum*, ano with idioblast (black arrow). (**G**) *A. patens*, sta with sinuous ACW. (**H**) *A. sieboldii*. (**I**) *A. versicolor*. (**H**,**I**) ano with sinuous ACW. (**J**) *Houttuynia cordata*, sta with idioblast (black arrow) and with glandular trichomes (white arrow). (**K**) *Piper kadsura*, ani and tet. (**L**) *Saururus chinensis*, act. (J–L) polar rods to the guard cells and fusiform slit ledge aperture. ano, anomocytic stomata; ani, anisocytic stomata; sta, staurocytic stomata; tet, tetracytic stomata; and ACW, anticlinal cell walls. Scale bars = 20 μm.

**Figure 3:**
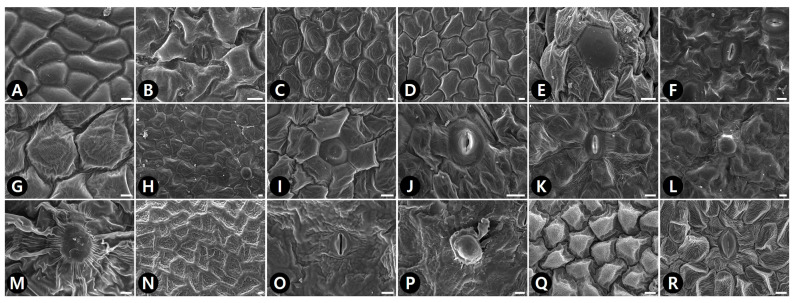
SEM micrographs of leaf epidermal characteristics with epidermal cells, stomatal complex, and secretory idioblast in Korean Piperales. (**A**) *Aristolochia contorta* (AD), convex PCW with smooth FR. (**B**) *A. contorta* (AB), unstriated stomatal surface with smooth guard cells. (**C**) *Asarum heterotropoides* var. *mandshuricum* (AD), conical PCW with striate and wrinkled FR. (D–E) *A. heterotropoides* var. *seoulense* (AD). (**D**) Convex PCW with striate and wrinkled FR. (**E**) Polygonal secretory idioblast with flat surface. (**F**) *A. heterotropoides* var. *seoulense* (AB), radiating striated stomatal surface and concentric rings guard cells. (**G**) *A. koreanum* (AD), convex to conical PCW with striate and wrinkled FR. (**H**) *A. maculatum* (AD), striate and wrinkled FR, and hexagonal secretory idioblast with flat surface. (**I**) *A. misandrum* (AD), hexagonal secretory idioblast with central tubercle surface. (**J**) *A. misandrum* (AB), lip-shaped stomatal ledge with radiating striated stomatal surface and concentric rings guard cells. (**K**) *A. patens* (AB), lip-shaped stomatal ledge with radiating striated stomatal surface and concentric rings guard cells. (**L**) *A. versicolor* (AB), secretory idioblast with convex surface. (**M**) *Houttuynia cordata* (AB), circular secretory idioblast with flat surface. (**N**) *Piper kadsura* (AD), penta- to polygonal cell with tuberculate FR, thick cell walls. (O–P) *P. kadsura* (AB). (**O**) Lip-shaped stomatal ledge with striations extended as lateral wings and concentric rings guard cells. (**P**) Circular secretory idioblast with protruding surface. (**Q**) *Saururus chinensis* (AD), conical PCW with striate and wrinkled FR. (**R**) *S. chinensis* (AB), lip-shaped stomatal ledge with radiating striations and smooth guard cells. ACW, anticlinal cell walls; FR, fine relief; and PCW, Periclinal cell wall. Scale bars = 10 μm.

The epidermal cell patterns were separately described for adaxial (AD) and abaxial (AB) leaf surfaces. Leaf epidermal cells were arranged isodiametrically or irregularly, and their shapes were penta-, hexa- to polygonal, irregular to polygonal, or irregular (Table S1). The isodiametric epidermal cells usually had straight or straight to curved anticlinal walls (ACW), whereas irregular cells usually had undulous or sinuous ACW (Table S1; Figure 1, Figure 2 and Figure 3). Straight ACW were found on both surfaces in *Aristolochia manshuriensis* (Figure 1A), *Piper kadsura* (Figure 1K and Figure 2K and *Saururus chinensis* (Figure 1L and Figure 2L). The periclinal cell wall (PCW) of all taxa on the AB surface were convex; however, for most taxa the PCW on the AD surface were convex or convex to conical (Table S1). For *P. kadsura* the PCW on the AD surface were only concave, and for *S. chinensis* the PCW on the AD surface were only conical. In most taxa, the epidermis was either smooth and not striated or wrinkled with parallel or radiating cuticular striation (Table S1). In contrast, *P. kadsura* had a tuberculate and wrinkled epidermis (Figure 1K and Figure 3N).

### 2.2. Idioblast Cells

Epidermal secretory idioblasts were found on all studied taxa except *Aristolochia*. The epidermis had penta-, hexa- to polygonal, or circular idioblasts (Figure 1D,G,H and Figure 2A,D,F,J,K). Furthermore, their surface patterns were flat (Figure 3E,H,M) to convex (Figure 3L), central tubercle (Figure 3I), or protruding (Figure 3P). The protruding secretory idioblasts were only found in *P. kadsura* (Figure 3P).

### 2.3. Trichomes

**Figure 4:**
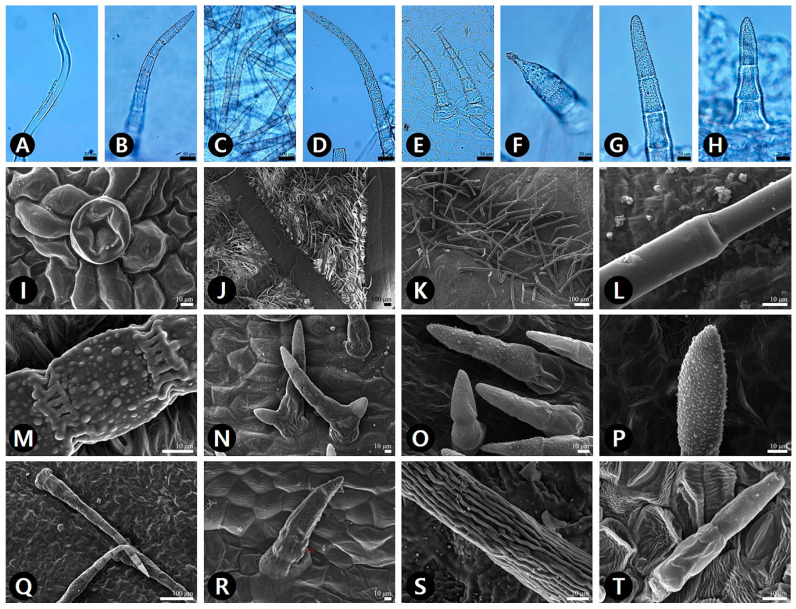
LM and SEM micrographs of trichomes on the leaf surface in the studied taxa. (**A**,**J**–**L**,**T**) sNT (smooth simple multicellular non-glandular trichomes). (**B**–**H**,**M**,**O**–**R**) vNT (verrucate simple multicellular non-glandular trichomes). (**I**) GT (glandular trichomes). (**N**) vYT (verrucate two-armed Y-shaped multicellular non-glandular trichomes). (**S**) tNT (striate simple multicellular non-glandular trichomes). (**A**) *Aristolochia manshuriensis* (AB). (**B**) *Asarum heterotropoides* var. *mandshuricum* (AB). (**C**) *A. heterotropoides* var. *seoulense* (AB). (**D**) *A. heterotropoides* var. *seoulense* (AD). (**E**) *A. koreanum* (AB). (**F**) *A. misandrum* (AD). (**G**,**H**) *A. sieboldii* (AB). (I) *Aristolochia contorta* (AB). (**J**) *A. manshuriensis* (AB). (**K**,**L**) *A. manshuriensis* (AD). (**L**) Smooth surface. (**M**) *A. heterotropoides* var. *seoulense* (AB), verrucate surface. (**N**) *A. koreanum* (AD). (**O**) *A. misandrum* (AD). (**P**,**Q**) *A. patens* (AB). (**R**) *A. sieboldii* (AD). (**S**) *Piper kadsura* (AB). (**T**) *Saururus chinensis* (AB).

**Figure 5:**
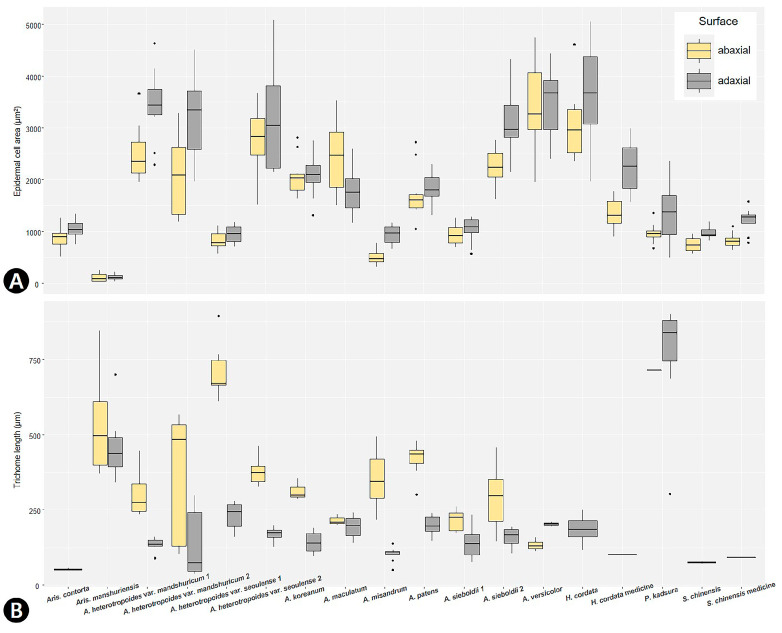
The size of epidermal characteristics with epidermal cells and trichomes in Korean Piperales. Grouped boxplots show the median, 25th and 75th percentiles (box), 10th and 90th percentiles (whiskers), and outliers (closed circle). (**A**) Actual epidermal cell area (μm^2^). (**B**) Trichome length (μm^2^).

The leaf epidermis of all studied species except *Saururus* was covered by hairs of various types (Table S1). Both surfaces of *S. chinensis* were glabrous (Figure 1L and Figure 2L). Three main types of trichome were defined—simple multicellular non-glandular trichomes (NT), two-armed multicellular non-glandular Y-shaped trichome (YT), and glandular trichomes (GT) (Table S1; Figure 4). Four subtypes of non-glandular trichomes were observed, based on the surface patterns and shapes—smooth NT (sNT) (Figure 4L,T), striate NT (tNT) (Figure 4S), verrucate NT (vNT) (Figure 4M,P), and verrucate two-armed Y-shaped NT (vYT) (Figure 4N). The vNT was found on both surfaces of all taxa of *Asarum* (Table S1). Notably, the AB surface of two varieties of *A. heterotropoides* was moderate to densely pubescent with vNT. The epidermis of *Aristolochia manshuriensis* on the AB surface was invisible because of a dense layer of sNT (Figure 4J). The sNT were usually distributed on the main vein of the AD surface of *H. cordata*. The tNT was only found on the AB surface of *P. kadsura* (Figure 4S). The peltate GT was present in the following taxa—on the AB surface of *A. contorta* (Figure 4I), *Asarum heterotropoides* var. *mandshuricum*, *A. heterotropoides* var. *seoulense*, and *P. kadsura*; and on the AD surface of *H. cordata* (Table S1). The longest trichomes were found on the AB surface of *P. kadsura* (mean ± S.D., 761 ± 185 μm), and the shortest trichomes were found on the AB surface of *Aristolochia contorta* (53 ± 2 μm). In the case of pubescence on both surfaces, the length of trichome was longer on the AB surface than on the AD surface (Figure 5B).

### 2.4. Stomatal Complex

Amphistomatic leaves were found in the genera *Asarum* and *Houttuynia*, whereas the hypostomatic leaves were found in the *Aristolochia*, *Piper*, and *Saururus* (Table S2). Five types of stomatal complexes were recognized—actinocytic, anisocytic, anomocytic, tetracytic, and staurocytic. The most common type of stomatal complex in the taxa investigated was anomocytic. However, two or three types of stomata occurred on the same surface. An actinocytic stomatal complex was observed in *S. chinensis* (Figure 2L). Stomatal ledges were either only lip-shaped or lip-shaped and double semicircle. Stomata with thick and wide outer stomatal ledge aperture usually had a linear slit in *Aristolochia contorta* (Figure 2A). In contrast, those with narrowly elliptical ledges usually possessed elliptic slits in *A. manshuriensis* and all taxa in the genus *Asarum*. Moreover, stomata with polar rods to the guard cells ledge aperture had a fusiform slit in the genera *Houttuynia*, *Piper*, and *Saururus* (Table S2). The surface of the guard cells was either smooth (*Aristolochia* and *Saururus*) or had concentric rings (*Asarum*, *Houttuynia*, and *Piper*). Three types of stomatal surface were defined—no-striae, striae extended as a lateral wing, and radiating striae.

**Figure 6:**
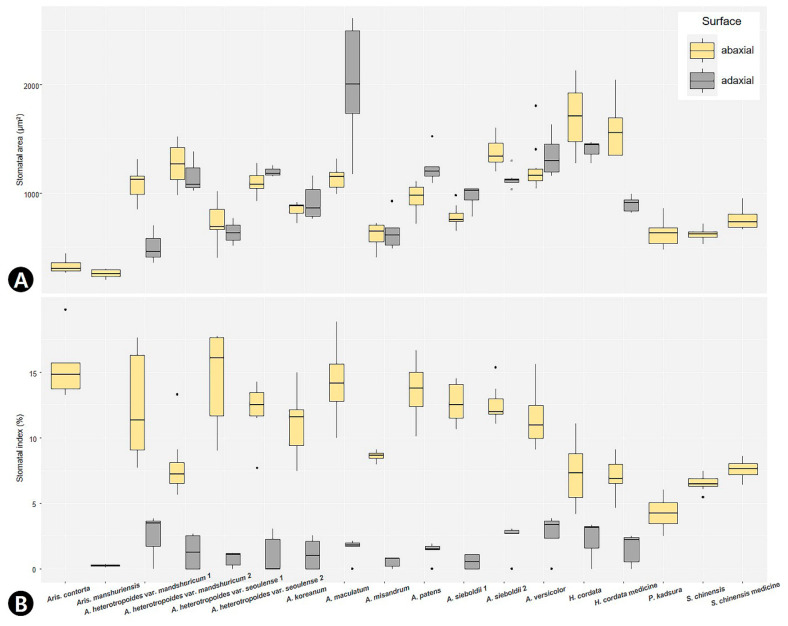
The quantitative data of stomatal characteristics with the stomatal area and stomatal index in Korean Piperales. Grouped boxplots show the median, 25th and 75th percentiles (box), 10th and 90th percentiles (whiskers), and outliers (closed circle). (**A**) Actual stomatal complex area (μm^2^). (**B**) Stomatal index (%).

The smallest stoma was recorded in *Aristolochia manshuriensis* on the AB surface (mean ± S.D.: 256 ± 34 μm^2^), and the largest stoma was recorded in *Asarum maculatum* on the AD surface (2005 ± 524 μm^2^) (Table S4). In the case of amphistomatic leaves, the stomatal area (SCA) was larger on the AB surface than on the AD surface, but some species had a larger SCA on the AD surface (Figure 6A). SCA was significantly positively correlated with the epidermal cell actual area (ECA) (*r* = 0.528, *P* < 0.001). The highest stomatal density (SD) was observed on the AB surface of *A. heterotropoides* var. *seoulense* 1 leaves (17.40 ± 3.38 cells), and the lowest SD was observed on the AB surface of *Aristolochia manshuriensis* leaves (2.00 ± 0.71 cells) (Table S4). The stomatal index (SI) of all amphistomatic taxa was higher on the AB surface than on the AD surface (Figure 6B). On the AD surface, the highest SI was observed in *Asarum versicolor* (2.64 ± 1.54%), and the lowest SI was observed in *A. patens* (0.15 ± 0.08%). On the AB surface, the highest SI was recorded in *Aristolochia contorta* (15.46 ± 2.33%), and the lowest SI was recorded in *A. manshuriensis* (2.00 ± 0.71%). SD was significantly positive correlated with SI (*r* = 0.8119, *P* < 0.001). Potential conductance index (PCI) was always higher for the AB surface than for the AD surface. The highest PCI was recorded for *Asarum sieboldii* 2 (2.21 ± 0.95), and the lowest PCI for *A. heterotropoides* var. *mandshuricum* 1 (0.05 ± 0.04) and var. *seoulense* 1 (0.05 ± 0.05) (Table S5).

### 2.5. Identification Key Based on Leaf Micromorphology

Based on the leaf epidermal micromorphological analysis, a key was designed for accurate identification and authentication of the Korean Piperales.
1. Stomatal position amphistomatic ........................................................................................................... 2 1’. Stomatal position hypostomatic ............................................................................................................ 3 2. Stomatal ledge aperture polar rods to the guard cells; stomatal pore fusiform slit ..................................................................................................................................... *Houttuynia cordata* 2’. Stomatal ledge narrowly elliptical; stomatal pore elliptic slit ........................................................................................................................................................................ 4 3. Stomatal ledge aperture polar rods to the guard cells; stomatal pore fusiform slit; stomatal surface striae extended as lateral wing or radiating striae ..................................................................... 5 3’. Stomatal ledge aperture narrowly elliptical; stomatal pore elliptic slit; stomatal surface not striated ........................................................................................................................................................... 6 4. Presence of both glandular trichomes and verrucate multicellular non-glandular trichomes on the abaxial surface .......................................... *Asarum heterotropoides* vars. *mandshuricum* and *seoulense* 4’. Presence of verrucate multicellular non-glandular trichomes only on the abaxial surface ............................................................................................................................................................... 7 5. Stomatal surface striae extended as lateral wing; stomatal types anisocytic and tetracytic; guard cells concentric rings; secretory idioblasts protruding ........................................................ *Piper kadsura* 5’. Stomatal surface radiating striae; stomatal type actinocytic; guard cells smooth; secretory idioblasts flat to central tubercle .................................................................................... *Saururus chinensis* 6. Fine relief of the epidermal cells smooth; sparsely pubescent with glandular trichomes on the abaxial surface; stomatal ledge aperture thick and wide outer stomatal ledge with linear slit pore ................................................................................................................................. *Aristolochia contorta* 6’. Fine relief of the epidermal cells striate and wrinkled; densely pubescent with verrucate multicellular non-glandular trichomes on the abaxial surface; stomatal ledge aperture narrowly elliptical with elliptic slit pore ........................................................................... *Aristolochia manshuriensis* 7. Stomatal types anomocytic, staurocytic, and tetracytic ...................................................................... 8 7’. Stomatal type anomocytic only ............................................................................................................. 9 8. Secretory idioblasts flat to convex; presence of verrucate Y-shaped multicellular non-glandular trichomes ........................................................................................................................... *Asarum koreanum* 8’. Secretory idioblasts flat to central tubercle; absence of verrucate Y-shaped multicellular non- glandular trichomes ................................................................................................................................... 10 9. Undulous anticlinal cell walls on the adaxial surface; fine relief of the epidermal cells on the abaxial surface smooth .................................................................................................. *Asarum maculatum* 9’. Straight to curved anticlinal cell walls on the adaxial surface; fine relief of the epidermal cells on the abaxial surface striate and wrinkled ............................................................................................ 11 10. Straight to curved anticlinal cell walls on the abaxial surface; sparsely pubescent with verrucate multicellular non-glandular trichomes on the abaxial surface ................................ *Asarum misandrum* 10’. Sinuous anticlinal cell walls on the abaxial surface; densely pubescent with verrucate multicellular non-glandular trichomes on the abaxial surface ........................................ *Asarum patens* 11. Irregular epidermal cell arrangement and presence of pentagonal secretory idioblasts on the adaxial surface; sparsely pubescent with verrucate multicellular non-glandular trichomes and presence of double semi-circular stomatal ledge on the abaxial surface ................................................................................................................................ *Asarum versicolor* 11’. Isodiametric epidermal cell arrangement and absence of pentagonal secretory idioblasts on the adaxial surface; moderate to densely pubescent with verrucate multicellular non-glandular trichomes and only presence of lip-shaped stomatal ledge on the abaxial surface ................................................................................................................................... *Asarum sieboldii*

## 3. Discussion

Using the results of LM and SEM microscopic analysis, we found useful and reliable identification characteristics, and developed a leaf micromorphological identification key that distinguishes all Korean Piperales taxa.

### 3.1. Leaf Micromorphology and Its Taxonomic Implication in Korean Piperales

This study was the first comprehensive approach using leaf cuticular morphology covering all genera of Korean Piperales, although leaf micromorphology for certain species and genera have been reported in previous studies [55,56,57,58]. The leaf characteristics of Korean Piperales show a great diversity in epidermal cell patterns, stomatal complex, and structure of idioblasts and trichomes.

Previous studies have suggested that the pattern of ACW might be closely related to their habitat environment, e.g., straight to curved ACW are representative of species from drier habitats, whereas undulous to sinuous ACW are representative of species from more humid habitats [59]. However, in the current study straight or straight to curved ACW appeared in species occurring in humid and wet habitats, including *Houttuynia cordata* and *Saururus chinensis*. Thus, our data do not support this hypothesis. The most studied taxa had convex PCW with striate and wrinkled FR; however, *Piper kadsura* exhibited a unique pattern, which is concave PCW with tuberculate and thick cell walls on the AD surface. As a result of this, *P. kadsura* was easily distinguished from the other taxa.

The secretory idioblasts are considered to be oil-secreting cells in Piperaceae [60,61]. In particular, morpho-anatomical, ontogenic, or histochemical studies have been conducted in selected taxa of the genus *Piper* [55,61] and *Houttuynia cordata* [62]. Although further studies are required to clarify the anatomical/internal structures and their contained compounds, we found that the presence and types of secretory idioblasts were useful characteristics for the identification of taxa in Korean Piperales.

The presence or absence of trichomes and their density are affected by various ecological factors [63]. Nevertheless, the trichomes on leaves of certain taxa in Piperales have been used for diagnostic purposes from a macro-morphological point of view [29,64,65,66,67]. Furthermore, we evaluated that the trichome types and their surface patterns are useful for the delimitation and identification among taxa included in the present study. All sampled taxa of *Asarum* had verrucate-surfaced NT, which might be considered to be a generic synapomorphy. The smooth-surfaced NT were only found on the AD surface of *H. cordata* and both surfaces of *Aristolochia manshuriensis*. Additionally, striate surfaced NT were only found on the AB surface of *P. kadsura*. Of the seven species of *Asarum*, only the two varieties of *A. heterotropoides* had GT on the AB surface, and only *A. koreanum* had verrucate-surfaced YT on the AD surface. Thus, the surface patterns of trichomes might be useful diagnostic characteristics for species as well as genus identification.

For the Korean taxa, the stomatal position was consistently observed at the genus level and considered to be a useful diagnostic characteristic. However, in contrast to hypostomatic Korean *Piper*, amphistomatic leaves were also reported in *P. leptostachyon* Nutt. [68], *P. hispidinervum* C. DC. [69], and *P. sarmentosum* Roxb. [70,71]. The amphistomatic stomata position is generally characteristic of species occupying xerophytic habitats [72]. However, *Piper* including *P. sarmentosum* is mainly distributed in the tropical or subtropical region [73]. Therefore, this cannot be assumed to be a characteristic feature of xerophytes only.

The anomocytic stomatal type was most common, but staurocytic and tetracytic stomata were found together with anomocytic stomata in some taxa. Moreover, anisocytic stomata were also found together with staurocytic (*H. cordata*) or tetracytic stomata (*P. kadsura*), whereas actinocytic stomata were not found with other types of stomata (*S. chinensis*). Thus, the patterns of stomatal type in the studied group might represent a useful diagnostic characteristic. Other stomatal characteristics, such as the stomatal ledge apertures and the surface of the stomatal complex in Lauraceae, have also proved to be useful diagnostic characteristics [44]. We also found that a combination of the stomatal characteristics with the ledge apertures, pore shapes, and the surface of the stomatal complex were useful in discriminating between the Korean Piperales in this study, and the shapes of the ledge aperture were closely related to the pore shapes. *Houttuynia*, *Piper*, and *Saururus* shared the same ledge aperture and pore; however, each genus could be well distinguished based on the stomata and guard cell surfaces.

SD and SI are highly influenced by the environmental factors; e.g., CO_2_ levels [74] and light intensities [75]. Consequently, high stomatal frequency is related to habitat, and high light intensities play a role in increasing photosynthesis and transpiration rates [76]. In the hypostomatic *Aristolochia*, *A. contorta*, which is usually found in sunny areas at the base of mountains, had a high average SD (16.80 counts/mm^2^) and SI (15.46%), whereas *A. manshuriensis*, which is usually found in shady areas and moist valleys, had a low average SD (2.00 counts/mm^2^) and SI (0.21%). With respect to ecophysiology, Driscoll et al. [77] suggested that the photosynthesis and transpiration rates were always higher on the AB surface than on the AD surface. In all amphistomatic taxa studied, SI was always higher and not overlapping on the AB than on the AD surface. Thus, SD and SI could be considered to be an indirect indicator for the degree of photosynthesis and transpiration rates.

The cell size of plant organs is determined by the degree of polyploidy, genome size, phytohormone signaling, and developmental factors [78,79]. In the present study, most of the quantitative epidermal and stomatal characteristics were variable, even on the same surface of the same taxa; this was especially the case with respect to ECA with ranges of values of 2.35–7.62 times, SCA with ranges of values of 1.62–3.93 times, SD with ranges of values of 2.66–5.26 times, and SI with ranges of values of 1.44–3.85 times. Although several researchers have used quantitative data for developing the taxonomic key [6,10,80], a more careful application is needed to distinguish the taxa.

### 3.2. Leaf Micromorphology for Taxonomy of Korean Asarum

Although various new taxa and their distribution have been reported in previous studies [81,82,83,84], the species and infraspecific relationships and the delimitation of the Korean *Asarum* are still controversial. In the taxonomic revision of the genus, So and Kim [35] defined three species and seven forma, whereas Oh [37] suggested seven separate species. We followed Oh’s classification [37], which recognized species level, and evaluated his system using leaf micromorphological characteristics.

Although the trichome density of the AB surface is influenced by the habitat environment, the trichome characteristics are useful to discriminate certain taxa. Moreover, all *Asarum* taxa studied shared the type of vNT, and thus this could be considered as a synapomorphic characteristic of the *Asarum*. The vNTs were only found on margins of *A. maculatum* leaves (not on the laminal part). The two varieties of *A. heterotropoides* and *A. patens* have longer trichomes (average length 438 μm and 420 μm, respectively) than other taxa (average length 140 μm).

So and Kim [35] recognized *A. heterotropoides* as two infraspecies based on the presence or absence of trichomes on the petiole. In contrast, Oh [37] treated the infraspecific *seoulense* as synonymous to *A. mandshuricum* and elevated it to the species level. In the present study, *A. heterotropoides* var. *mandshuricum* and var. *seoulense* mostly shared common leaf micromorphological characters and these could not be used in the taxonomic key to distinguish between the two varieties. Moreover, we found a clinal variation on the petiole between glabrous and pubescent *A. heterotropoides* taxa. Although further cytological study is required to understand the correlation with epidermal and stomatal cell size variation and polyploidy within this group, the present leaf epidermal characteristics support the taxonomic concept of Oh [37]. A combination of an external morphological key [37] and the leaf micromorphological key presented here could provide an accurate way to discriminate among Korean *Asarum* taxa.

### 3.3. Application for Authentication of Herbal Medicine

Piperales in Korea includes ‘Asiasari Radix et Rhizoma’, ‘Piperis Kadsurae Caulis’, ‘Houttuyniae Herba’, and ‘Saururi Herba’ as herbal medicines [31]. However, several misuse issues have emerged because of the distribution and sale of mixed, adulterated, or counterfeit medicinal materials [85,86]. Misuse of traditional medicines might result in severe harm to patients [87]. Thus, recent attempts have been made to improve the authentication of medicinal material using microscopic analysis [88,89,90]. Our studied character states revealed that the collected and purchased materials were identical and overlapping. This data matrix, which includes the qualitative characteristics of correctly identified taxa, gives us objective evidence to identify the medicinal materials. Thus, leaf micromorphological characteristics and statistical techniques might be useful tools for more accurate and effective authentication of aerial plant parts used as a medicine.

## 4. Materials and Methods

### 4.1. Taxon Sampling and Identification

To observe the leaf morphological characteristics of the Korean Piperales, including five genera mature leaves of 13 taxa and 18 accessions were used. The plant materials used in this study were mainly collected from natural populations. Some materials were taken from herbarium specimens obtained from the ‘Collection and Investigation of National Medicinal Herbs Project’. All studied species were identified and confirmed based on major diagnostic characteristics mentioned by the Flora of Korea [29]. Notably, in the genus *Asarum*, we followed Oh’s taxonomic concept [37] except for *A. mandshuricum* (Maxim.) M. Kim and S. So. In the case of *A. mandshuricum*, we recognized two varieties (vars. *mandshuricum* (Maxim.) Kitag. and *seoulense* (Nakai) Kitag.) based on the presence or absence of trichomes on petioles, in order to verify their taxonomic status.

In the case of *Houttuynia cordata* Thunb. and *Saururus chinensis* (Lour.) Baill. whose aerial parts are used as a medicine and are known as ‘Houttuyniae Herba’ and ‘Saururi Herba,’ respectively, samples were purchased in Korean herbal medicinal markets from commercial suppliers. All collected and purchased medicinal materials were deposited in the Korean Herbarium of Standard Herbal Resources (KIOM) at the Korea Institute of Oriental Medicine, Naju, Korea (Table S6).

### 4.2. Light Microscopic Analysis

Prior to observing the leaf epidermal characteristics, all dried leaf samples (which were taken from herbarium specimens) were first examined using an Olympus SZX16 stereomicroscope (Olympus, Tokyo, Japan), in order to select fully mature leaves. Fragments (1 to 2 cm^2^) were taken from the middle region of leaves. For each plant species, two to three samples were prepared. The dried materials were rehydrated overnight in a wetting agent Agepon^®^ (Agepon: distilled water, 1:200) (Agfa Gevaert, Leverkusen, Germany). The leaf samples were immersed in a tube filled with 5% sodium hypochlorite (NaClO) at room temperature for three to five hours, until the samples started to whiten. The samples were then washed in water. The epidermis of both the adaxial (AD) and abaxial (AB) surfaces of leaves were peeled off and on to a microscope slide. The slides were then examined using an Olympus BX-53 light microscope (Olympus, Tokyo, Japan), and images were captured using a digital camera (Olympus DP21, Olympus, Tokyo, Japan).

### 4.3. Scanning Electron Microscopic Analysis

To observe the more detailed epidermal characteristics such as trichome types and surfaces, and cuticular and stomatal striation, the samples were rehydrated in wetting agent and then washed in 70% ethyl alcohol; the leaves were then fixed in FAA solution (40% formalin: 40% glacial acetic acid: 70% ethyl alcohol) for 24 h. The leaf samples were dehydrated through a graded ethanol series (50%, 70%, 90%, 95%, and 100% ethanol) at room temperature for one hour per ethanol concentration. The dehydrated materials were immersed in liquid carbon dioxide (CO_2_) for critical point drying (CPD, SPI-13200JE-AB, SPI Supplies, West Chester, PA, USA). The dried leaves were mounted on the aluminum stubs using a double-sided adhesive conductive carbon disk (05073-BA, SPI Supplies, West Chester, PA, USA). All samples were coated with gold using an ion-sputtering device (208HR; Cressington Scientific Instruments Ltd., Watford, UK), and all samples were observed using a low-voltage field emission scanning electron microscope (JSM-7600F, JEOL, Tokyo, Japan) at an accelerating voltage of 5–10 kV with a working distance of 8–10 mm.

### 4.4. Quantitative Data Analysis

The quantitative characteristics were analyzed using different quantitative measures, on both AD and AB surfaces; e.g., epidermal cell length (ECL), epidermal cell width (ECW), cell wall width (CWW), epidermal cell actual area (ECA), epidermal cell density (ECD), and trichome length (TL), (Table S3); stomatal complex length (SCL), stomatal complex width (SCW), stomatal complex area (SCA), stomatal density (SD), stomatal index (SI), and potential conductance index (PCI) (Table S4). Additional quantitative data were also provided; subsidiary cell length (SBL), subsidiary cell width (SBW), subsidiary cell actual area (SBA), stomatal ledge aperture length (SAL), stomatal ledge aperture width (SAW), and stomatal ledge aperture actual area (SAA) (Table S5).

All quantitative data of epidermal cells and stomata complex obtained from LM and SEM images were determined from the Digimizer software (Digimizer version 5.4.3, MedCalc Software, Mariakerke, Belgium). The SI value was calculated as described by Salisbury [91]: SI = S/E + S × 100, where SI = Stomatal index, S = Number of stomata per unit area (mm^2^), and E = Number of epidermal cells per unit area (mm^2^). The PCI value was calculated as described by Holland and Richardson [92]: PCI = (SCL)^2^ × SD × 10^−4^.

To demonstrate variations in ECA, TL, SCA, and SI, both the AD and AB surfaces among the species grouped boxplots were generated using the ggplot2 library [93] in R package version 3.6.3 (R Foundation for Statistical Computing, Vienna, Austria).

The terminology of leaf micromorphological characteristics followed those of Stace [1], Metcalfe and Chalk [2], Yang et al. [43], and Esau [94].

## 5. Conclusions

We fully described the leaf micromorphology of all Korean Piperales taxa. A comprehensive taxonomic consideration of the characteristics, such as epidermal surfaces, stomata types and positions, trichome types and density, and secretory idioblast types proved that these were useful additional diagnostic characteristics. Quantitative epidermal and stomatal characteristics, which are affected by environmental factors, were not effective diagnostic characteristics because of the considerable variation within the same taxa. Among the studied characteristics, especially, stomata type, surface and the types of trichome and density, provided additional taxonomic evidence and supported previous taxonomic revision. Furthermore, our study demonstrated that the comprehensive investigation of leaf micromorphological characteristics using microscopic and statistical analysis is a reliable approach for accurate discrimination between authentic herbal medicines and counterfeit or adulterated material.

## Supplementary Materials

Supplementary materials can be found at https://www.mdpi.com/2223-7747/9/5/566/s1. Table S1. Qualitative characteristics of leaves epidermal cells, idioblasts, and trichomes in Korean Piperales. Table S2. Qualitative characteristics of stomatal complex in Korean Piperales. Table S3. Quantitative epidermal characteristics in Korean Piperales; Min (Mean ± S.D.) Max. Table S4. Quantitative stomatal characteristics in Korean Piperales; Min (Mean ± S.D.) Max, Table S5. Quantitative subsidiary cells and stomatal ledge aperture in Korean Piperales; Min (Mean ± S.D.) Max. Table S6. Voucher specimens of Korean Piperales examined in the present study.
