# Supplementary material for: Taxonomic Implications of Leaf Micromorphology Using Microscopic Analysis: A Tool for Identification and Authentication of Korean Piperales
Source: Plants (Basel). 2020 Apr 29;9(5):566. doi: 10.3390/plants9050566 (PMC7285214; doi:10.3390/plants9050566)
Supplement: Supplementary file 1 [file plants-09-00566-s001.pdf]

SUPPLEMENTARY MATERIALS

Taxonomic Implication of Leaf Micromorphology using Microscopic Analysis: A Tool for Identification and Authentication of Korean Piperales

Jun-Ho Song, Sungyu Yang and Goya Choi*

Herbal Medicine Reseources Research Center, Korea Institute of Oriental Medicine, Naju, 58245, Republic of Korea

* Correspondence: Telephone: +82-61-338-7118; Fax: +82-61-338-7135; E-mail addresses: songjh@kiom.re.kr (J.-H.S.); sgyang81@kiom.re.kr (S.Y.); serparas@kiom.re.kr (G.C.)

Table S1. Qualitative characteristics of leaves epidermal cells, idioblasts, and trichomes in Korean Piperales.

Taxa	Epidermal cell					Secretory idioblast		Trichome	
	Arrangement	Shape	Anticlinal cell wall (ACW)	Periclinal cell wall (PCW)	Fine relief (FR)	Shape	Surface	Presence	Type
Aristolochia									
A. contorta (AD)	iso	irr to pol	stg to cur	cvx	smt	-	-	-	-
A. contorta (AB)	irr	irr	sin	cvx	smt	-	-	+	GT
A. manshuriensis (AD)	iso	pen, hex to pol	stg	cvx	str, wrk	-	-	+	sNT
A. manshuriensis (AB)	iso	pen, hex to pol	stg	cvx	str, wrk	-	-	+++	sNT
Asarum									
A. heterotropoides var. *mandshuricum* 1 (AD)	iso	irr to pol	und	cvx to con	str, wrk	hex to pol	flt to cvx	++	vNT
A. heterotropoides var. *mandshuricum* 1 (AB)	irr	irr	sin	cvx	str, wrk	hex to pol	flt to cvx	++	vNT, GT
A. heterotropoides var. *mandshuricum* 2 (AD)	iso	irr to pol	und	cvx to con	str, wrk	hex to pol	flt to cvx	++	vNT
A. heterotropoides var. *mandshuricum* 2 (AB)	irr	irr	sin	cvx	str, wrk	hex to pol	flt to cvx	+++	vNT, GT
A. heterotropoides var. *seoulense* 1 (AD)	iso	irr to pol	und	cvx to con	str, wrk	hex to pol	flt to cvx	++	vNT
A. heterotropoides var. *seoulense* 1 (AB)	irr	irr to pol	und	cvx	str, wrk	hex to pol	flt to cvx	+++	vNT, GT
A. heterotropoides var. *seoulense* 2 (AD)	irr	irr to pol	und	cvx	str, wrk	hex to pol	flt to cvx	++	vNT
A. heterotropoides var. *seoulense* 2 (AB)	irr	irr to pol	und	cvx	str, wrk	hex to pol	flt to cvx	++	vNT, GT
A. koreanum (AD)	iso	irr to pol	stg to cur	cvx to con	str, wrk	hex to pol	flt to cvx	+	vNT, vYT
A. koreanum (AB)	irr	irr	sin	cvx	str, wrk	hex to pol	flt to cvx	+	vNT
A. maculatum (AD)	iso	irr to pol	und	cvx	str, wrk	hex to pol	flat to ctb	+	vNT (margin)
A. maculatum (AB)	irr	irr	sin	cvx	smt	hex to pol	flat to ctb	+	vNT (margin)
A. misandrum (AD)	iso	irr to pol	stg to cur	cvx	str, wrk	pen to hex	flat to ctb	++	vNT
A. misandrum (AB)	iso	irr to pol	stg to cur	cvx	str, wrk	pen to hex	flat to ctb	+	vNT
A. patens (AD)	iso	irr to pol	und	cvx to con	str, wrk	pen to hex	flat to ctb	+	vNT
A. patens (AB)	irr	irr	sin	cvx	str, wrk	pen to hex	flat to ctb	++	vNT
A. sieboldii 1 (AD)	iso	irr to pol	stg to cur	cvx	str, wrk	hex to pol	flt to cvx	+	vNT
A. sieboldii 1 (AB)	irr	irr	sin	cvx	str, wrk	hex to pol	flt to cvx	++	vNT
A. sieboldii 2 (AD)	iso	irr to pol	stg to cur	cvx	str, wrk	hex to pol	flt to cvx	++	vNT
A. sieboldii 2 (AB)	irr	irr	sin	cvx	str, wrk	pen to hex	flt to cvx	++	vNT
A. versicolor (AD)	irr	irr to pol	stg to cur	cvx	str, wrk	pen to hex	flt to cvx	+	vNT

A. versicolor (AB)	irr	irr	sin	cvx	str, wrk	pen to hex	flt to cvx	+	vNT
Houttuynia									
H. cordata (AD)	iso	pen, hex to pol	stg	cvx	str, wrk	pol or cir	flt	+	GT, sNT (vein)
H. cordata (AB)	iso	irr to pol	stg to cur	cvx	smt	pol or cir	flt	-	-
H. cordata (AD)*	iso	pen, hex to pol	stg	cvx	str, wrk	pol or cir	flt	+	GT, sNT (vein)
H. cordata (AB)*	iso	irr to pol	stg to cur	cvx	str, wrk	pol or cir	flt	-	-
Piper									
P. kadsura (AD)	iso	pen, hex to pol	stg	ccv	tub	pol or cir	pro	-	-
P. kadsura (AB)	iso	pen, hex to pol	stg	cvx	tub, wrk	pol or cir	pro	+	tNT, GT
Saururus									
S. chinensis (AD)	iso	pen, hex to pol	stg	con	str, wrk	hex to pol	flat to ctb	-	-
S. chinensis (AB)	iso	pen, hex to pol	stg	cvx	str, wrk	hex to pol	flat to ctb	-	-
S. chinensis (AD)*	iso	pen, hex to pol	stg	con	str, wrk	hex to pol	flat to ctb	-	-
S. chinensis (AB)*	iso	pen, hex to pol	stg	cvx	str, wrk	hex to pol	flat to ctb	-	-

Epidermal cell arrangement: irr, irregular; iso, isodiametiric. *Shape*: hex, hexagonal; pen, pentagonal; pol, polygonal. *Anticlinal cell wall*: cur, curved; sin, sinuous; stg, straight; und, undulous. *Periclinal cell wall*: ccv, concave; con, conical; cvx, convex. *Fine relief*: smt, smooth; str, striate; tub, tuberculate; wrk, wrinkled. *Secretory idioblast shape*: cir, circle. Surface: ctb, central tubercle; flt, flat. *Trichomes type*: GT, glandular trichomes; NT, multi-cellular non-glandular trichomes; YT, Y-shaped multi-cellular non-glandular trichomes. *Trichome surface*: s, smooth; t, striate; v, verrucate. -, absent; +, present sparsely; ++, present moderately; +++, present densely. * Medicinal materials.

Table S2. Qualitative characteristics of stomatal complex in Korean Piperales.

Taxa	Position	Type	Stomatal ledge	Ledge aperture	Pore shape	Guard cells	Stomatal surface
Aristolochia							
A. contorta (AD)	Hypo	/	/	/	/	/	/
A. contorta (AB)		ano	lip	two	lins	smt	NS
A. manshuriensis (AD)	Hypo	/	/	/	/	/	/
A. manshuriensis (AB)		ano	lip, dsc	nel	ells	smt	NS
Asarum							
A. heterotropoides var. *mandshuricum* 1 (AD)	Amph	ano	lip, dsc	nel	ells	ccr	EW, RS
A. heterotropoides var. *mandshuricum* 1 (AB)		ano	lip, dsc	nel	ells	ccr	EW, RS
A. heterotropoides var. *mandshuricum* 2 (AD)	Amph	ano	lip, dsc	nel	ells	ccr	EW, RS
A. heterotropoides var. *mandshuricum* 2 (AB)		ano	lip, dsc	nel	ells	ccr	EW, RS
A. heterotropoides var. *seoulense* 1 (AD)	Amph	ano	lip, dsc	nel	ells	ccr	EW, RS
A. heterotropoides var. *seoulense* 1 (AB)		ano	lip, dsc	nel	ells	ccr	EW, RS
A. heterotropoides var. *seoulense* 2 (AD)	Amph	ano	lip, dsc	nel	ells	ccr	EW, RS
A. heterotropoides var. *seoulense* 2 (AB)		ano	lip, dsc	nel	ells	ccr	EW, RS
A. koreanum (AD)	Amph	ano, sta, tet	lip	nel	ells	ccr	RS
A. koreanum (AB)		ano, sta, tet	lip, dsc	nel	ells	ccr	RS
A. maculatum (AD)	Amph	ano	lip	nel	ells	ccr	RS
A. maculatum (AB)		ano	lip	nel	ells	ccr	RS
A. misandrum (AD)	Amph	ano, sta, tet	dsc	nel	ells	ccr	RS
A. misandrum (AB)		ano, sta, tet	lip, dsc	nel	ells	ccr	RS
A. patens (AD)	Amph	ano, sta, tet	lip, dsc	nel	ells	ccr	RS
A. patens (AB)		ano, sta, tet	lip, dsc	nel	ells	ccr	RS
A. sieboldii 1 (AD)	Amph	ano	lip	nel	ells	ccr	RS
A. sieboldii 1 (AB)		ano	lip	nel	ells	ccr	RS
A. sieboldii 2 (AD)	Amph	ano	lip	nel	ells	ccr	RS
A. sieboldii 2 (AB)		ano	lip	nel	ells	ccr	RS
A. versicolor (AD)	Amph	ano	lip	nel	ells	ccr	RS
A. versicolor (AB)		ano	lip, dsc	nel	ells	ccr	RS
Houttuynia							
H. cordata (AD)	Amph	ani, sta	lip	prg	fuss	ccr	RS
H. cordata (AB)		ani, sta	lip, dsc	prg	fuss	ccr	RS

H. cordata (AD)*	Amph	ani, sta	lip	prg	fuss	ccr	RS
H. cordata (AB)*		ani, sta	lip, dsc	prg	fuss	ccr	RS
Piper							
P. kadsura (AD)	Hypo	/	/	/	/	/	/
P. kadsura (AB)		ani, tet	lip	prg	fuss	ccr	EW
Saururus							
S. chinensis (AD)	Hypo	/	/	/	/	/	/
S. chinensis (AB)		act	lip	prg	fuss	smt	RS
S. chinensis (AD)*	Hypo	/	/	/	/	/	/
S. chinensis (AB)*		act	lip	prg	fuss	smt	RS

Position: Amph, amphistomatic; Hypo, hypostomatic. *Type*: act, actinocytic; ano, anomocytic; ani, anisocytic; sta, staurocytic; tet, tetracytic. *Stomatal ledge*: dsc, double semicircle; lip, lip-shaped. *Ledge aperture*: nel, narrowly elliptical; prg, polar rods to the guard cells; two, thick and wide outer stomatal ledge. *Pore shape*: ells, elliptic slit; fuss, fusiform slit; lins, linear slit. *Guard cells*: ccr, concentric rings; smt, smooth. *Stomatal surface*: NS, no-striae; EW, striae extended as lateral wing; RS, radiating striae. /, not applicable. * Medicinal materials.

Table S3. Quantitative epidermal characteristics in Korean Piperales. Min(Mean±S.D.)Max.

Taxa	Epidermal cell					Trichome
	Length (ECL, μm)	Width (ECW, μm)	Actual area (ECA, μm²)	Cell wall width (CWW, μm)	Cell density (ECD, counts/mm²)	Length (TL, μm)
Aristolochia						
A. contorta (AD)	35.67(41.80 ± 5.08)53.04	20.23(25.12 ± 3.34)29.94	751(1045 ± 158)1334	1.08(1.87 ± 0.41)2.67	90(95 ± 4.26)102	-
A. contorta (AB)	23.84(36.36 ± 8.45)49.84	12.84(23.88 ± 8.41)41.76	526(876 ± 190)1266	0.30(1.07 ± 0.39)1.79	81(92 ± 7.39)102	51(53 ± 2.75)57
A. manshuriensis (AD)	6.33(12.58 ± 4.17)18.18	7.08(8.73 ± 1.50)12.18	47(122 ± 52)223	0.80(1.08 ± 0.18)1.31	888(943 ± 47.12)1012	342(454 ± 97.89)700
A. manshuriensis (AB)	5.07(12.28 ± 5.09)20.65	4.94(8.67 ± 3.01)12.94	38(116 ± 81)257	0.30(1.12 ± 0.45)1.81	890(944 ± 51.10)1021	371(522 ± 141.93)844
Asarum						
A. heterotropoides var. *mandshuricum* 1 (AD)	58.53(82.26 ± 13.4)101.6	31.80(44.04 ± 7.08)56.71	2284(3452 ± 656)4627	1.23(2.02 ± 0.53)3.28	25(28 ± 2.87)32	91(135 ± 22.97)162
A. heterotropoides var. *mandshuricum* 1 (AB)	56.75(71.20 ± 8.39)84.00	19.22(39.08 ± 10.87)56.12	1956(2487 ± 513)3657	1.19(2.35 ± 0.66)3.59	42(48 ± 4.30)55	237(308 ± 83.86)448
A. heterotropoides var. *mandshuricum* 2 (AD)	56.04(76.41 ± 14.17)98.67	33.51(44.47 ± 7.57)59.66	1968(3174 ± 802)4510	1.19(2.18 ± 0.45)2.69	31(36 ± 3.03)40	37(141 ± 106.07)300
A. heterotropoides var. *mandshuricum* 2 (AB)	41.61(58.96 ± 11.74)78.20	16.79(26.51 ± 7.02)41.93	1184(2048 ± 710)3282	1.19(1.99 ± 0.61)3.07	35(43 ± 5.27)52	105(365 ± 197.64)568
A. heterotropoides var. *seoulense* 1 (AD)	22.23(37.48 ± 6.76)44.77	17.46(27.07 ± 4.52)34.10	715(953 ± 157)1180	1.49(2.00 ± 0.43)2.70	79(86 ± 4.57)93	163(229 ± 42.50)280
A. heterotropoides var. *seoulense* 1 (AB)	25.32(34.35 ± 7.91)54.66	17.74(23.50 ± 3.44)29.91	569(838 ± 173)1118	0.90(1.85 ± 0.56)2.55	88(104 ± 11.5)121	610(702 ± 79.13)892
A. heterotropoides var. *seoulense* 2 (AD)	57.70(80.01 ± 17.60)106.7	29.42(43.56 ± 8.83)56.12	2152(3144 ± 933)5077	1.51(1.84 ± 0.23)2.21	32(41 ± 5.05)47	129(170 ± 19.05)200
A. heterotropoides var. *seoulense* 2 (AB)	46.37(63.99 ± 11.27)84.60	24.48(36.46 ± 10.47)58.48	1513(2726 ± 634)3669	1.61(2.39 ± 0.41)2.98	42(46±1.99)48	329(378 ± 42.48)462
A. koreanum (AD)	49.43(62.16 ± 8.24)74.61	26.35(34.60 ± 6.94)49.38	1312(2092 ± 400)2747	0.90(1.62 ± 0.42)2.39	38(45 ± 4.45)50	98(143 ± 34.92)191
A. koreanum (AB)	46.58(62.14±8.63)74.87	29.91(38.87 ± 6.32)50.13	1629(2076 ± 363)2812	0.67(1.84 ± 0.69)3.40	51(59 ± 4.76)65	287(314 ± 30.26)356
A. maculatum (AD)	39.56(57.99 ± 11.92)83.70	22.08(36.10±6.24)43.91	1165(1768 ± 401)2597	2.98(3.82 ± 0.60)5.38	46(53 ± 5.00)60	142(193 ± 34.19)242
A. maculatum (AB)	39.69(66.88 ± 14.91)91.18	13.26(35.13 ± 11.85)51.69	1506(2422 ± 637)3529	1.47(2.58 ± 0.46)3.35	41(42 ± 1.34)45	201(216 ± 15.28)237
A. misandrum (AD)	29.51(35.34 ± 5.64)50.04	20.29(27.50 ± 3.86)34.16	665(938 ± 167)1168	1.19(2.13 ± 0.52)2.98	116(125 ± 5.63)132	51(104 ± 22.24)140
A. misandrum (AB)	20.05(28.53 ± 6.90)39.85	13.01(16.86 ± 3.55)25.50	321(506 ± 148)780	1.19(1.67 ± 0.30)2.17	128(177 ± 29.24)200	218(352 ± 85.78)495
A. patens (AD)	45.27(58.16±7.27)69.59	30.77(35.19 ± 2.88)41.03	1318(1815 ± 297)2289	1.79(2.23 ± 0.37)2.75	51(58 ± 5.31)65	148(200 ± 31.71)240
A. patens (AB)	38.60(51.46 ± 9.10)68.41	18.40(35.02 ± 10.84)53.67	1041(1718 ± 478)2721	0.67(1.73 ± 0.62)3.12	66(74 ± 6.22)83	301(420 ± 48.15)480
A. sieboldii 1 (AD)	33.73(45.34 ± 6.58)53.98	16.54(25.13 ± 4.37)30.78	568(1032 ± 234)1285	1.30(1.63 ± 0.28)2.32	81(88 ± 5.02)99	78(141 ± 46)235
A. sieboldii 1 (AB)	31.35(42.24 ± 8.57)62.44	14.56(22.74 ± 3.56)26.71	708(942 ± 187)1271	0.92(1.38 ± 0.29)1.82	88(92 ± 3.07)98	173(215 ± 30)262

A. sieboldii 2 (AD)	44.52(70.10 ± 13.56)90.73	41.60(46.72 ± 5.41)60.22	2149(3109 ± 631)4330	2.35(2.95 ± 0.35)3.25	32(34 ± 1.58)36	107(159 ± 30.76)194
A. sieboldii 2 (AB)	47.54(67.74 ± 9.49)82.98	28.66(37.13 ± 4.84)45.71	1625(2262 ± 344)2768	1.54(2.29 ± 0.64)3.44	44(46 ± 3.06)52	146(293 ± 100)458
A. versicolor (AD)	66.66(84.36 ± 12.3)104.55	39.5(49.40 ± 6.16)65.59	2400(3489 ± 629)4437	1.47(2.51 ± 0.82)4.41	20(26 ± 3.96)31	196(203 ± 6.18)210
A. versicolor (AB)	46.07(77.87 ± 16.32)107.9	33.44(52.89 ± 13.49)74.99	1955(3438 ± 847)4735	2.33(2.97 ± 0.68)4.56	27(32 ± 3.24)37	115(134 ± 18.18)159
Houttuynia						
H. cordata (AD)	65.80(83.67 ± 13.31)104.72	28.50(45.88 ± 10.57)59.65	1963(3699 ± 980)5048	1.08(2.17 ± 0.76)3.40	22(27 ± 3.10)31	117(185 ± 39.70)250
H. cordata (AB)	70.97(84.07 ± 12.16)107.34	29.78(43.90 ± 10.33)63.06	2366(3068 ± 640)4601	1.88(2.69 ± 0.70)4.21	20(25 ± 3.87)33	-
H. cordata (AD)*	38.53(59.28 ± 11.17)81.56	36.42(46.21 ± 8.97)66.51	1573(2227 ± 463)2989	1.32(2.03 ± 0.45)2.66	39(42 ± 2.15)46	83(115 ± 21.82)142
H. cordata (AB)*	40.31(51.39 ± 6.21)60.16	26.21(33.14 ± 5.38)42.47	901(1353 ± 267)1773	1.21(1.89 ± 1.70)2.57	40(41 ± 1.70)44	-
Piper						
P. kadsura (AD)	30.62(50.12 ± 14.21)78.70	20.57(28.07 ± 6.50)41.28	499(1411 ± 588)2363	3.61(4.92 ± 0.84)6.38	62(73 ± 5.50)80	-
P. kadsura (AB)	36.13(41.45 ± 3.21)46.24	19.19(26.53 ± 5.13)36.52	675(963 ± 179)1356	1.91(2.69 ± 0.45)3.38	99(109 ± 6.20)118	304(761 ± 185)898
Saururus						
S. chinensis (AD)	27.68(33.98 ± 4.12)39.56	23.58(30.18 ± 4.70)40.33	825(964 ± 109)1189	1.33(2.10 ± 0.39)2.70	106(110 ± 3.09)115	-
S. chinensis (AB)	24.22(35.33 ± 6.26)44.44	17.41(22.01 ± 3.52)29.10	579(755 ± 142)956	0.94(1.66 ± 0.41)2.33	102(113 ± 6.68)122	-
S. chinensis (AD)*	26.55(30.10 ± 2.16)34.45	27.53(39.30 ± 7.58)56.04	782(1213 ± 223)1576	1.18(1.85 ± 0.49)2.60	71(76 ± 3.81)81	-
S. chinensis (AB)*	18.66(32.63 ± 8.16)43.29	23.63(33.33 ± 9.76)47.00	649(829 ± 132)1101	1.52(1.89 ± 0.18)2.12	73(87 ±9 .00)99	-

* Medicinal materials.

Table S4. Quantitative stomatal characteristics in Korean Piperales. Min(Mean±S.D.)Max.

Taxa	Stomatal complex					Potential conductance index (PCI)
	Length (SCL, μm)	Width (SCW, μm)	Stomatal area (SCA, μm²)	Stomatal density (SD, counts/mm²)	Stomatal index (SI, %)	
Aristolochia						
A. contorta (AD)	-	-	-	-	-	-
A. contorta (AB)	21.32(23.03 ± 1.84)26.92	15.86(17.57 ± 1.56)21.39	261(324 ± 59)444	14(16.80 ± 2.32)20	13.27(15.46 ± 2.33)19.80	0.69(0.92 ± 0.15)1.16
A. manshuriensis (AD)	-	-	-	-	-	-
A. manshuriensis (AB)	18.16(19.58 ± 1.34)22.09	15.35(17.37 ± 1.88)20.59	201(256 ± 34)302	1(2.00 ± 0.71)3	0.11(0.21 ± 0.08)0.33	0.03(0.08 ± 0.04)1.15
Asarum						
A. heterotropoides var. *mandshuricum* 1 (AD)	23.35(26.18 ± 2.53)29.49	18.84(25.13 ± 5.01)31.11	352(503 ± 145)700	0(0.67±0.47)1	0.00(2.43±1.73)3.85	0.00(0.05±0.04)0.09
A. heterotropoides var. *mandshuricum* 1 (AB)	36.15(42.08 ± 2.95)45.88	29.94(34.32 ± 2.36)38.69	847(1087 ± 133)1310	4(6.83 ± 2.27)10	7.69(12.39 ± 3.98)17.65	0.82(1.26 ± 0.43)1.89
A. heterotropoides var. *mandshuricum* 2 (AD)	43.48(45.70 ± 1.88)48.07	35.17(36.76 ± 1.59)38.36	1020(1161 ± 159)1384	0(0.30 ± 0.46)1	0.00(1.28 ± 1.29)2.70	0.00(0.07 ± 0.10)0.23
A. heterotropoides var. *mandshuricum* 2 (AB)	40.57(45.50 ± 3.35)52.36	31.69(36.14 ± 2.66)40.15	982(1267 ± 182)1518	3(3.60 ± 0.92)6	5.66(7.77 ± 2.10)13.33	0.49(0.75 ± 0.22)1.17
A. heterotropoides var. *seoulense* 1 (AD)	28.69(30.93 ± 1.33)32.22	23.69(27.04 ± 2.17)29.25	512(634 ± 96)768	0(0.67 ± 0.47)1	0.00(0.75 ± 0.53)1.18	0.00(0.05 ± 0.05)0.10
A. heterotropoides var. *seoulense* 1 (AB)	22.07(31.25 ± 5.55)42.50	19.11(25.67 ± 2.94)30.08	402(733 ± 168)1015	12(17.40 ± 3.38)21	9.02(14.45 ± 3.49)17.76	1.83(2.11 ± 0.25)2.56
A. heterotropoides var. *seoulense* 2 (AD)	46.87(47.66 ± 0.57)48.20	33.24(34.10 ± 0.72)35.01	1150(1196 ± 43)1254	0(0.38 ± 0.48)1	0.00(0.98 ± 1.28)3.03	0.00(0.09 ± 0.11)0.23
A. heterotropoides var. *seoulense* 2 (AB)	36.47(40.89 ± 2.93)44.48	27.81(33.13±2.43)36.58	925(1090 ± 108)1278	4(6.29 ± 1.16)8	7.69(12.10 ± 2.02)14.29	0.53(1.06 ± 0.29)1.57
A. koreanum (AD)	38.61(42.42 ± 3.42)46.80	26.32(33.16 ± 6.91)45.77	765(920 ± 152)1159	0(0.50 ± 0.50)1	0.00(1.11 ± 1.12)2.56	0.00(0.09 ± 0.09)0.22
A. koreanum (AB)	34.16(38.19 ± 2.19)40.70	28.32(30.72 ± 1.04)32.23	725(851 ± 61)918	5(7.40 ± 1.62)9	7.46(11.12 ± 2.56)15.00	0.70(1.08 ± 0.30)1.45
A. maculatum (AD)	46.83(54.63 ± 4.54)60.04	36.19(48.04 ± 8.63)58.86	1173(2005 ± 524)2612	0(0.60 ± 0.49)1	0.00(1.60 ± 0.67)2.13	0.00(0.15 ± 0.15)0.36
A. maculatum (AB)	41.86(46.97 ± 3.40)53.99	28.91(31.78 ± 1.47)33.60	989(1139 ± 111)1320	5(7.17 ± 1.57)10	10.0(14.28 ± 2.77)18.87	1.29(1.57 ± 0.38)2.35
A. misandrum (AD)	25.86(29.28 ± 2.49)32.53	23.46(25.93 ± 2.40)29.85	484(636 ± 148)923	0(0.67 ± 0.47)1	0.00(0.52 ± 0.37)0.83	0.00(0.06 ± 0.04)0.11
A. misandrum (AB)	26.16(32.09 ± 4.65)38.46	19.63(24.80 ± 2.61)28.50	404(613 ± 105)726	12(16.75 ± 3.11)20	8.00(8.60 ± 0.40)9.09	1.13(2.09 ±0 .77)2.96
A. patens (AD)	40.28(42.89 ± 2.76)48.15	35.34(36.81 ± 1.08)38.02	1090(1242 ± 148)1522	0(0.80 ± 0.40)1	0.00(0.15 ± 0.08)0.23	0.00(1.33 ± 0.68)1.92
A. patens (AB)	28.07(36.56 ± 3.56)40.58	28.81(31.92 ± 1.78)34.19	716(959 ± 114)1108	8(11.75 ± 2.86)15	10.13(13.60 ± 2.36)16.67	1.25(1.71 ± 0.47)2.39
A. sieboldii 1 (AD)	34.34(37.38 ± 2.69)42.09	28.89(31.73 ± 2.59)35.70	786(962 ± 95)1036	0(0.50 ± 0.50)1	0.00(0.54 ± 0.54)1.12	0.00(0.07 ± 0.07)0.18
A. sieboldii 1 (AB)	30.58(36.90 ± 4.15)42.21	22.95(27.90 ± 4.71)36.49	653(780 ± 88)978	11(13.43 ± 1.92)16	10.68(12.70 ± 1.44)14.55	1.29(1.86 ± 0.37)2.35
A. sieboldii 2 (AD)	43.31(47.38 ± 1.87)49.02	29.62(32.29 ± 2.06)35.89	1031(1130 ± 75)1299	0(0.88 ± 0.33)1	0.00(0.20 ± 0.08)0.24	0.00(1.72 ± 0.20)2.05

A. sieboldii 2 (AB)	44.86(49.83 ± 3.07)55.09	31.32(35.44 ± 2.88)40.30	1199(137 ± 134)1599	6(6.71 ± 0.70)8	11.11(12.59 ± 1.36)15.38	1.35(2.21 ± 0.95)3.03
A. versicolor (AD)	50.09(52.11 ± 2.25)55.79	30.19(32.99 ± 2.07)35.25	1160(1346 ± 186)1633	0(0.75 ± 0.43)1	0.00(2.64 ± 1.54)3.85	0.00(0.21 ± 0.12)0.31
A. versicolor (AB)	36.69(45.25 ± 4.27)54.79	29.97(34.10 ± 2.95)39.88	1039(1231 ± 213)1804	3(4.17 ± 0.69)5	9.09(11.54 ± 2.18)15.53	0.73(0.88 ± 0.17)1.20
Houttuynia						
H. cordata (AD)	51.44(52.66 ± 0.87)53.46	33.88(35.91 ± 2.06)38.74	1275(1395 ± 85)1470	0(0.67 ± 0.47)1	0.00(2.15 ± 1.52)3.33	0.00(0.19 ± 0.13)0.29
H. cordata (AB)	45.80(53.68 ± 5.53)62.56	35.72(39.53 ± 3.37)47.39	1277(1690 ± 271)2129	1(2.00 ± 0.71)3	4.17(7.34 ± 2.31)11.11	0.24(0.58 ± 0.27)1.14
H. cordata (AD)*	35.01(37.54 ± 2.04)40.22	27.62(30.79 ± 2.18)33.41	820(898 ± 64)995	0(0.70 ± 0.46)1	0.00(1.62 ± 1.06)2.50	0.00(0.07 ± 0.07)0.16
H. cordata (AB)*	38.02(43.91 ± 3.69)48.48	37.99(42.89 ± 3.65)49.66	1287(1568 ± 244)2045	2(3.14 ± 0.64)4	4.65(7.04 ± 1.42)9.09	0.43(0.59 ± 0.13)0.84
Piper						
P. kadsura (AD)	-	-	-	-	-	-
P. kadsura (AB)	26.80(29.27 ± 1.78)32.16	23.00(26.01 ± 1.96)30.21	478(622 ± 110)863	3(4.80 ± 1.17)7	2.52(4.25 ± 1.05)6.03	0.30(0.41 ± 0.09)0.60
Saururus						
S. chinensis (AD)	-	-	-	-	-	-
S. chinensis (AB)	29.84(32.14 ± 1.81)34.99	23.80(24.92 ± 1.27)28.35	530(618 ± 54)718	7(7.90 ± 0.83)9	5.47(6.53 ± 0.54)7.50	0.62(0.82 ± 0.11)1.07
S. chinensis (AD)*	-	-	-	-	-	-
S. chinensis (AB)*	30.93(35.80 ± 2.54)39.13	25.11(27.83 ± 1.88)30.70	665(753 ± 86)953	5(7.20 ± 0.98)9	6.41(7.61 ± 0.66)8.57	0.67(0.92 ± 0.16)1.21

* Medicinal materials.

Table S5. Quantitative subsidiary cells and stomatal ledge aperture in Korean Piperales. Min(Mean±S.D.)Max.

Taxa	Subsidiary cells			Stomatal ledge aperture		
	Length (SBL, μm)	Width (SBW, μm)	Area (SBA, μm²)	Length (SAL, μm)	Width (SAW, μm)	Area (SAA, μm²)
Aristolochia						
A. contorta (AD)	-	-	-	-	-	-
A. contorta (AB)	31.09(37.03 ± 5.25)49.16	17.36(25.40 ± 7.38)14.47	568(894 ± 200)1266	15.34(19.80 ± 2.36)24.34	6.54(8.50 ± 1.48)12.25	78(115 ± 23)152
A. manshuriensis (AD)	-	-	-	-	-	-
A. manshuriensis (AB)	5.67(8.81 ± 1.33)10.46	4.49(8.12 ± 3.33)13.88	43(69 ± 27)135	6.45(8.70 ± 1.95)11.04	3.07(4.93 ± 1.53)7.78	3(33 ± 18)55
Asarum						
A. heterotropoides var. *mandshuricum* 1 (AD)	65.29(81.85 ± 15.09)106.59	41.53(50.23 ± 8.51)62.53	3507(3901 ± 362)4560	14.73(14.79 ± 0.06)14.85	8.44(9.13 ± 0.70)9.83	87(105 ± 18)123
A. heterotropoides var. *mandshuricum* 1 (AB)	49.11(69.12 ± 13.53)91.21	23.81(37.91±7.39)50.72	1623(2464 ± 459)3291	17.04(22.70 ± 3.05)26.56	6.87(10.86 ± 1.71)12.89	98(186 ± 43)265
A. heterotropoides var. *mandshuricum* 2 (AD)	29.65(47.38 ± 11.52)57.61	21.00(33.24 ± 13.44)54.68	714(1655 ± 685)2586	24.35(25.36 ± 0.95)26.63	9.57(11.80 ± 1.58)12.99	148(198 ± 37)237
A. heterotropoides var. *mandshuricum* 2 (AB)	13.73(24.14 ± 6.88)38.42	13.85(34.12 ± 10.46)53.16	574(1050 ± 326)1796	17.91(24.10 ± 3.61)31.10	10.19(11.47 ± 1.08)13.28	121(197 ± 79)397
A. heterotropoides var. *seoulense* 1 (AD)	24.45(41.12 ± 9.61)56.57	13.21(27.01 ± 8.91)40.99	198(741 ± 330)1328	9.52(17.68 ± 7.07)26.25	4.54(6.28 ± 1.11)7.46	32(65 ± 22)94
A. heterotropoides var. *seoulense* 1 (AB)	23.55(38.10 ± 9.93)55.70	12.69(20.27 ± 5.39)28.42	372(878 ± 293)1238	9.28(17.69 ± 4.99)22.96	4.44(7.37 ± 2.01)10.38	23(108 ± 64)220
A. heterotropoides var. *seoulense* 2 (AD)	22.68(32.87 ± 7.41)46.47	16.23(28.08 ± 10.94)48.50	426(848 ± 393)1752	21.92(23.76 ± 2.60)27.43	7.34(9.46 ± 1.55)11.00	127(149 ± 24)183
A. heterotropoides var. *seoulense* 2 (AB)	22.30(30.94 ± 6.65)41.79	18.97(28.19 ± 7.36)42.68	522(932 ± 384)1600	17.07(21.08 ± 2.39)24.98	7.24(8.46 ± 0.85)10.52	80(131 ± 24)169
A. koreanum (AD)	22.54(27.66 ± 5.63)36.35	19.47(25.25 ± 4.01)30.42	300(560 ± 166)752	16.46(18.89 ± 2.26)21.89	7.67(9.21 ± 1.00)10.44	85(126 ± 22)144
A. koreanum (AB)	15.16(26.54 ± 4.62)31.60	13.40(22.71 ± 7.28)36.16	395(685 ± 193)1028	16.58(19.70 ± 1.90)22.71	8.90(10.93 ± 1.82)14.28	108(147 ± 24)184
A. maculatum (AD)	26.70(31.26 ± 3.18)35.88	15.67(37.60 ± 13.66)61.42	627(1050 ± 337)1569	18.38(25.70 ± 4.47)31.34	10.61(14.17 ± 2.70)18.24	222(306 ± 65)369
A. maculatum (AB)	20.96(31.69 ± 8.80)52.08	23.14(34.46 ± 8.31)49.69	861(1233 ± 234)1582	22.67(25.33 ± 1.58)28.65	10.65(11.74 ± 0.79)13.05	177(238 ± 29)285

A. misandrum (AD)	9.66(21.14 ± 7.37)31.34	7.75(19.20 ± 10.22)32.70	117(341 ± 125)510	8.44(10.42 ± 1.94)14.04	4.22(6.38 ± 1.16)7.46	37(53 ± 12)70
A. misandrum (AB)	15.02(21.04 ± 5.37)33.66	9.64(12.98 ± 3.44)21.19	123(249 ± 124)578	10.98(17.84 ± 4.38)24.97	6.33(8.05 ± 1.20)10.28	55(100 ± 29.6)142
A. patens (AD)	25.07(36.89 ± 11.77)63.58	22.68(47.40 ± 15.01)69.98	321(506 ± 148)780	20.55(21.60 ± 0.78)22.82	11.18(11.40 ± 0.18)11.70	156(184 ± 15)199
A. patens (AB)	18.61(27.65 ± 5.41)38.31	10.86(18.26 ± 6.44)27.80	760(1530 ± 440)2196	10.55(17.71 ± 3.74)24.32	6.89(9.38 ± 1.20)10.97	42(100 ± 36)167
A. sieboldii 1 (AD)	10.27(26.99 ± 9.22)40.42	13.74(19.94 ± 6.13)35.75	244(546 ± 164)847	10.60(14.94 ± 3.46)20.10	7.61(8.81 ± 1.03)10.50	66(101 ± 29)146
A. sieboldii 1 (AB)	17.61(32.80 ± 7.16)46.01	16.08(20.78 ± 3.28)27.10	508(736 ± 142)934	10.75(17.14 ± 4.97)24.67	7.49(9.38 ± 0.97)10.69	74(128 ± 47)218
A. sieboldii 2 (AD)	22.60(35.69 ± 9.30)49.82	20.29(43.50 ± 13.88)68.61	649(1673 ± 636)2573	22.55(24.06 ± 1.11)26.01	9.72(11.54 ± 2.00)16.08	202(224 ± 19)260
A. sieboldii 2 (AB)	25.26(31.77 ± 5.06)44.95	21.76(33.00 ± 10.59)55.39	698(1176 ± 569)2677	25.05(28.04 ± 1.66)30.82	10.52(13.03 ± 1.63)15.76	177(280 ± 48)348
A. versicolor (AD)	37.28(47.39 ± 7.24)57.70	29.04(42.16 ± 9.59)55.30	823(1537 ± 448)2062	22.58(26.13 ± 2.89)30.24	9.48(11.31 ± 1.95)14.58	172(200 ± 20)229
A. versicolor (AB)	17.66(32.82 ± 10.81)50.16	20.12(45.00 ± 82.27)18.44	653(1657 ± 709)2711	18.06(26.81 ± 3.99)33.98	8.36(12.05 ± 1.73)13.80	123(208 ± 58)325
Houttuynia						
H. cordata (AD)	16.28(39.53 ± 17.11)63.95	16.54(31.80 ± 11.27)55.01	655(965 ± 211)1338	38.74(40.57 ± 1.44)42.26	10.36(11.41 ± 1.41)13.41	195(286 ± 66)348
H. cordata (AB)	17.90(30.07 ± 10.10)48.72	27.67(48.66 ± 13.10)77.01	887(1421 ± 616)3156	38.91(44.47 ± 4.85)54.92	14.93(18.12 ± 2.80)25.32	266(440 ± 121)669
H. cordata (AD)*	15.91(29.46 ± 10.27)40.41	14.06(24.31 ± 9.81)41.56	521(638 ± 81)765	16.42(20.89 ± 2.61)23.79	5.59(8.81 ± 1.82)10.58	69(127 ± 31)162
H. cordata (AB)*	16.46(29.56±10.55)49.10	27.99(40.68±9.41)58.80	742(1007±175)1307	21.74(25.73±2.65)31.11	8.04(14.55 ± 3.27)18.34	87(246 ± 85)394
Piper						
P. kadsura (AD)	-	-	-	-	-	-
P. kadsura (AB)	13.71(28.50±7.45)34.47	12.16(17.72±6.60)35.88	291(423±90)613	22.95(25.20±1.68)28.17	6.38(8.52 ± 1.39)11.21	85(133 ± 39)204
Saururus						
S. chinensis (AD)	-	-	-	-	-	-
S. chinensis (AB)	15.27(20.10±3.18)24.66	13.09(20.86±4.06)28.43	304(429±71)555	18.32(23.69±4.08)30.35	6.18(8.48 ± 1.46)10.86	69(105 ± 26)153
S. chinensis (AD)*	-	-	-	-	-	-
S. chinensis (AB)*	16.81(23.80 ± 8.86)48.16	16.77(21.78 ± 2.83)25.80	350(462 ± 75)563	20.22(24.43 ± 2.80)30.83	5.85(8.23 ± 1.61)11.35	102(139 ± 21)173

* Medicinal materials.

Table S6. Voucher specimens of Korean Piperales that are examined in the present study.

Taxa	Voucher specimens
**Aristolochiaceae Juss.**	
***Aristolochia* L.**	
A. contorta Bunge	Yeongchun-myeon, Danyang-gun, Chungcheongbuk-do (37°02'43.3"N 128°36'21.4"E), B.C. Moon & S. Yang, 28. July 2016. KIOM201701018780
A. manshuriensis Kom.	Banwol-dong, Deokjin-gu, Jeonju-si, Jeollabuk-do, Y.S. Ju & H.J. Kim, 26. April 2009. KIOM200901002588
***Asarum* L.**	
A. heterotropoides F. Schmidt var. *mandshuricum* (Maxim.) Kitag. 1 [≡*A. mandshuricum* (Maxim.) M. Kim & S. So]	Hwayasan Mt., Cheongpyeong-myeon, Gapyeong-gun, Gyeonggi-do (37°41'36.4"N 127°24'13.9"E), J.-H. Song & S. Yang, 27. April 2018. KIOM201901022356
A. heterotropoides var. *mandshuricum* 2	Cheonmasan Mt., Hopyeong-dong, Namyangju-si, Gyeonggi-do (37°40'28.8"N 127°15'25.3"E), J.-H. Song & S. Yang, 28. April 2018. KIOM201901022351
A. heterotropoides var. *seoulense* (Nakai) Kitag. 1 [=*A. mandshuricum* (Maxim.) M. Kim & S. So]	Anchang-ri, Jijeong-myeon, Wonju-si, Gangwon-do (37°20'32.6"N 127°48'54.3"E), J.-H. Song & S. Yang, 26. April 2018. KIOM201901022340
A. heterotropoides var. *seoulense* 2	Cheonmasan Mt., Onam-eup, Namyangju-si, Gyeonggi-do (37°40'48.2"N 127°15'44.8"E), J.-H. Song & S. Yang, 28. April 2018. KIOM201901022349
A. koreanum J.G.Kim & C.S. Yook ex B.U. Oh	Geumsusan Mt., Sang-ri, Jeokseong-myeon, Danyang-gun, Chungcheongbuk-do (36°59'09.6"N 128°15'36.4"E), J.-H. Song & S. Yang, 26. April 2018. KIOM201901022336
A. maculatum Nakai	Albamaegioreum, Jochon-eup, Jeju Si, Jeju-Do, Y. Ji & B.C. Moon, 24. July 2012. KIOM201201004571
A. patens (Yamaki) M. Kim & S. So	Seondosan Mt., Sangdang-gu, Cheongju-si, Chungcheongbuk-do (36°37'50.7"N 127°33'24.7"E), J.-H. Song & S. Yang, 21. April 2018. KIOM201901022344
A. sieboldii Miq. 1	Wontongsa Temp., Anseong-myeon, Muju-gun, Jeollabuk-do, Y. Ji, 07. May 2013. KIOM201401009283
A. sieboldii 2	Chilgapsan Mt., Cheongyang-gun, Chungcheongnam-do, B.C. Moon, 04. June 2013. KIOM201401009286
A. versicolor	Bukgachi, Songnisan Mt., Boeun-gun, Chungcheongbuk-do (36°33'40.0"N 127°49'48.1"E), J.-H. Song & S. Yang, 25. April 2018. KIOM201901022368
**Saururaceae Rich. ex T. Lestib.**	
***Houttuynia* Thunb.**	
H. cordata Thunb.	San 58-3, Gagok-dong, Miryang-si, Gyeongsangnam-do (35°28'55.9"N 128°45'57.7"E), H.J. Choi, 03. May 2019. MFDS-V-11236<br>Andong-si technology center of GBA, Andong-si, Gyeongsangbuk-do (36°35'40.6"N 128°32'48.2"E), G. Choi, 03. July 2014. KIOM201501013841
H. cordata (Houttuyniae Herba)*	purchased in medicinal markets from commercial suppliers in South Korea (material management number 2-20-0055)
***Saururus* L.**	
S. chinensis (Lour.) Baill.	2410-1, Yongsu-ri, Hangyeong-myeon, Jeju-si, Jeju (33°19'11.6"N 126°10'49.8"E), J.H. Lee, 12. August 2019. MFDS-V-6846<br>Buk-myeon, Ulleung-gun, Gyeongsangbuk-do, Y. Ji & B.C. Moon, 19. June 2012. KIOM201201004718
S. chinensis (Saururi Herba)*	purchased in medicinal markets from commercial suppliers in South Korea (material management number 2-20-0054)
**Piperaceae Giseke**	

***Piper* L.**	
P. kadsura (Choisy) Ohwi	1410-3, Napeup-ri, Aewol-eup, Jeju-si, Jeju, Jeju (33°25'47.1"N 126°19'44.3"E), J.H. Lee, 12. October 2018. MFDS-V-194
